# Complete Genome Sequence Analysis and Probiotic Characterisation of *Lactiplantibacillus plantarum* Y300 Isolated from Traditional Free-Range Chickens

**DOI:** 10.3390/microorganisms13122738

**Published:** 2025-11-30

**Authors:** Xiaoyu Zhang, Xuehuai Shen, Dongdong Yin, Jieru Wang, Ruihong Zhao, Yin Dai, Erhui Jin, Xiaocheng Pan, Lei Yin

**Affiliations:** 1 College of Animal Science, Anhui Science and Technology University, Chuzhou 233100, China; 15563786108@163.com; 2 Anhui Provincial Key Laboratory of Livestock and Poultry Product Safety, Livestock and Poultry Epidemic Diseases Research Center of Anhui Province, Institute of Animal Husbandry and Veterinary Science, Anhui Academy of Agricultural Science, Hefei 230031, China; xuehuaishen1986@126.com (X.S.); yindd160@163.com (D.Y.); wangjr0317@126.com (J.W.); zrhkdy03@126.com (R.Z.); daiyin2020@163.com (Y.D.); pxcpyq@sina.com (X.P.)

**Keywords:** *Lactiplantibacillus plantarum*, safety, probiotic property, whole-genome sequencing, traditional free-range chickens

## Abstract

As a core strategy for antibiotic replacement, probiotics have two advantages insofar as they enhance both animal productivity and pathogen suppression. In this study, we screened the intestines of antibiotic-naïve chickens for broad-spectrum antimicrobial lactic acid bacteria (LAB) with natural adaptability, based on the host–microbiota coevolution theory, and systematically evaluated their potential for development as poultry probiotics. We isolated a LAB strain, *Lactiplantibacillus plantarum* Y300, from traditional native free-range chickens, which showed strong inhibitory activity against avian pathogenic *Salmonella*, *Escherichia coli*, and *Staphylococcus aureus*. In vitro experiments indicated that the *Lpb. plantarum* strain Y300 had no hemolytic activity; excellent acid-producing ability;an outstanding tolerance to bile salts, low-pH environments, and simulated gastrointestinal fluids; a positive hydrophobic interaction with xylene, and good auto-aggregation characteristics. It also displayed a relatively high antioxidant capacity. Whole-genome sequencing revealed that the genome of *Lpb. plantarum* Y300 was approximately 3.05 mb, with a GC content of 44.74%. The main carbohydrate-active enzyme and bacteriocin genes were predicted in the Y300 genome, and no virulence genes or drug-resistance genes were detected. In summary, this study suggests that *Lpb. plantarum* Y300 has potential utility as a probiotic, and lays the theoretical foundation for the further development of microecological preparations of avian-sourced LAB.

## 1. Introduction

The poultry industry is a cornerstone of the global animal protein supply, sustaining human nutritional needs, but faces escalating systemic risks from intensive production systems [1]. Multidrug-resistant pathogens, such as *Escherichia coli*, *Salmonella enterica*, and methicillin-resistant *Staphylococcus aureus* (MRSA), create bidirectional transmission pathways through contaminated food chains. This transmission is associated with substantial mortality on poultry farms and is responsible for over one million zoonotic infections in humans annually [2]. Rooted in six decades of the misuse of antibiotic feed additives, this crisis stems from a vicious cycle in which subtherapeutic antibiotic dosing induces conjugative-plasmid-mediated horizontal gene transfer, supporting superbugs carrying blaNDM-1 and other extended-spectrum β-lactamase genes. These resistance determinants accumulate in human gut reservoirs via food chains, triggering multidrug-resistant infections and microbiotal dysbiosis [3].

However, in this context, microbiome engineering is transforming the health management paradigm in animal husbandry. Probiotic systems centered around LAB have shown ecological value surpassing that of traditional antibiotics in that they construct a healthy defense network by imposing a triple biological barrier. Physically, LAB form biofilms via quorum sensing systems, competitively inhibiting the colonization of pathogens such as *Salmonella* [4,5]. Chemically, bacterial metabolites, such as bacteriocins and short-chain fatty acids, can penetrate pathogen biofilms, disrupting their quorum sensing systems [6,7]. Immunologically, they activate the TLR2/NF-κB signaling pathway, increasing secreted immunoglobulin A (sIgA) secretion and enhancing mucosal immune surveillance [8]. Recent studies have shown that specific functional strains offer targeted advantages. For instance, rhamnose-fermenting *Limosilactobacillus reuteri* enhances intestinal tight junctions by upregulating the expression of occludin protein, reducing *Salmonella* infection rates in broiler chickens by 67% [9,10]. *Limosilactobacillus acidophilus* simultaneously increases the daily weight gain and survival rates in broilers, by upregulating the expression of the host’s tight junction protein ZO-1 and significantly inhibiting the inflammatory cytokine interleukin 6 (IL6) [11], while also improving the survival rates of chickens infected with *E. coli* [12]. These data indicate that lactic acid bacteria play a crucial role in maintaining their hosts’ intestinal health and enhancing the efficiency of livestock husbandry. Thus, microbiome engineering is revolutionizing the management of poultry health.

Precision probiotic applications now dominate the innovation in this field. Metagenomics-guided fecal microbiotal transplantation reconstructs the core gut microbiome, and synbiotics enhance the abundance of *Bifidobacterium* 15-fold through probiotic–prebiotic synergy [13]. Clustered regularly interspaced short palindromic repeats (CRISPR)-engineered lactobacilli strains have achieved targeted pediocin production, specifically inhibiting *Listeria* with 93% efficacy while preserving the commensal microbiota [14]. These advances mark a paradigm shift from our reliance on chemicals to the precision control of microbes. However, identifying robust probiotics with enhanced stress resistance remains critical to optimizing animal health, production efficiency, and economic sustainability in modern poultry systems. In this study, we isolated *Lpb. plantarum* Y300 from local traditional native free-range chickens. A comprehensive analysis of the probiotic potential and safety of strain Y300 was undertaken with whole-genome sequencing and in vivo and in vitro tests, which demonstrated the strain’s potential utility in poultry farming.

## 2. Materials and Methods

### 2.1. Ethics Statement

The animal experiments were approved by the Institutional Animal Care and Use Committee at the Institute of Animal Husbandry and Veterinary Science, Anhui Academy of Agricultural Sciences (Permit No: AAAS-IAHVS-Po-2025-0819). The chickens were employed in experiments that complied with the ARRIVE guidelines of laboratory animal welfare and ethics [15].

### 2.2. Strain Isolation and Identification

*Lactiplantibacillus plantarum* Y300 was isolated from a 300-day-old, yellow-feathered broiler chicken raised under local traditional native free-range conditions. Under sterile conditions, the chicken cecum was excised, and the cecal contents carefully scraped out. A sample (1 g) of the cecal contents was added to 9 mL of 0.85% sterile saline and shaken at 37°C for 30 min. An appropriate amount (100 μL) of the suspension was spread on de Man–Rogosa–Sharpe (MRS) agar medium plates (with 2% CaCO_3_ added before sterilization). The plates were inverted and incubated at 37°C in a constant-temperature incubator for 24–48 h. Colonies with calcium-dissolving circles and different morphologies were picked and purified by streaking them multiple times. MRS broth (100 mL) was inoculated with purified single colonies, which were cultured at 37°C for 12 h, and then Gram stained. A 100× electron microscope (ECLIPSE Ci-L, Nikon Corporation, Tokyo, Japan) was used to observe and record the morphology of the strains. The isolate Y300 was identified as a lactic acid bacterium using biochemical test strips for carbohydrate fermentation. The bacterial DNA was then extracted with a bacterial genome extraction kit (Tiangen, Beijing, China). PCR was performed with primers 16S-27F (5-AGAGTTTGATCATGGCTCAG-3) and 16S-1492R (5-GGTTACCTTGTTACGACTT-3) [16]. The PCR-amplified products were sent to General Biosystems (Anhui) Co., Ltd. (Chuzhou, China) for sequencing. The 16S rDNA sequences obtained were compared with those in the National Center for Biotechnology Information database. The sequences with the greatest homology to Y300 were selected, and a phylogenetic tree was constructed with MEGA 7 software [17].

### 2.3. Safety Assessment

#### 2.3.1. Hemolytic Activity Assay

Columbia blood agar (Sangon Biotech Co., Ltd., Shanghai, China) was inoculated with each strain, which were then incubated for 24 h at 37 °C and checked for hemolysis. Strains with known hemolytic phenotypes were used as controls: *Staphylococcus aureus* subsp. *aureus* ATCC 25923. The hemolytic activity of the strains was detected as β-hemolysis (a clear, colorless/light yellow zone around the colony). In contrast, hemolytic inactivity was detected as α-hemolysis (a zone of greenish to brownish discoloration surrounding the colony) or γ-hemolysis (no change) [18].

#### 2.3.2. Antibiotic Susceptibility Testing

The antimicrobial susceptibility of the LAB was determined using the disc diffusion method. The selection of antimicrobial discs was performed in accordance with the European Food Safety Authority (EFSA) guidelines. Briefly [19], the bacterial suspension was adjusted to 10^8^ CFU/mL, and 100 µL aliquots were pipetted onto Mueller–Hinton (MH) agar plates. The bacteria were spread evenly and allowed to absorb for 10 min, after which antimicrobial susceptibility discs were gently placed on the agar surfaces with sterile forceps. The plates were incubated anaerobically at 37 °C for 24 h in a constant-temperature incubator. The diameters of the zones of inhibition were measured and recorded, with three replicates per test.

### 2.4. Characterization of Probiotic Properties

#### 2.4.1. Antimicrobial Activity Testing

The LAB isolates were cultured in MRS broth at 37 °C for 24 h, and the cell-free supernatant (CFS) was obtained by centrifugation at 6000× *g* for 10 min, followed by filtration sterilization using a sterilized 0.22 µm MILLEX^®^GP filter unit (PES membrane) (Merck Millipore, St. Louis, MO, USA).The Oxford cup agar diffusion method was used to evaluate the antimicrobial activity of the CFS of strain Y300 against various pathogens. Briefly [20], avian pathogenic *E. coli* (clinical isolate, strain ID: AE17), *Salmonella enterica Typhimurium* ATCC 14028, *Salmonella enterica Enteritidis* ATCC 13076, *Salmonella enterica Pullorum* CVCC79201, and *Staphylococcus aureus* subsp. *aureus* ATCC 25923 were used as indicator bacteria. Nutrient broth was inoculated separately with each pathogen, which were cultured at 37°C with agitation at 180 rpm for 8 h until they reached the logarithmic growth phase. The pathogenic bacterial suspensions were adjusted to 10^8^ CFU/mL, spread onto Luria–Bertani (LB) agar plates, and sterile cylinders (Oxford cups) were placed on the agar surface. The CFS of Y300 (200 μL) was added to each cylinder. After incubation at 37°C for 16–24 h, the diameters of the inhibition zones were measured and recorded, with three replicates per test.

#### 2.4.2. Growth and Acid Production Curves Tests

The growth and acid production of strain Y300 were monitored as previously described [21,22]. Briefly, an overnight culture of Y300 was prepared by incubating in MRS broth for 24 h. The cells were harvested by centrifugation, washed, and used to inoculate fresh MRS broth at 1% (*v*/*v*). The inoculated broth was incubated at 37 °C, and bacterial growth and acid production were monitored over time to generate the respective curves. An enzyme-linked immunosorbent assay (ELISA) reader was used to measure the optical density at a wavelength of 600 nm (OD600) of the bacterial culture every 4 h. Simultaneously, the pH was measured every 4 h with a pH detector (Shanghai INESA, Shanghai, China).

#### 2.4.3. Acid and Bile Tolerance Tests

Acid and bile salt tolerance were assessed as previously described [23]. The concentration of strain Y300 was adjusted to 10^8^ CFU/mL. A 2 mL sample of the prepared bacterial solution was centrifuged, the supernatant discarded, and the pellet resuspended in MRS liquid medium at pH 2.5 or pH 3.5, or in MRS liquid medium containing 0.3% or 0.4% porcine bile salts. All samples were incubated for 3 h. The bacterial solution (100 µL) was collected aseptically at 0 h (A_0_) and 3 h (A), spread onto plates containing MRS agar medium, and the colonies per plate counted. The experiment was performed with three replicates. The survival rate after acid or bile salt treatment = (A/A_0_) × 100%, where A_0_ is the viable cell count (CFU/mL) at 0 h (before treatment) and A is the viable cell count (CFU/mL) after 3 h of treatment.

#### 2.4.4. Tolerance of Simulated Gastric and Intestinal Fluids Tests

The tolerance of Y300 for simulated gastric and intestinal fluids was determined with the method reported by Sağlam et al. [24]. The concentration of strain Y300 was adjusted to 10^8^ CFU/mL. Samples (2 mL) of the prepared bacterial solution were centrifuged, the supernatant discarded, and the pellets resuspended in 2 mL of prepared artificial gastric fluid or artificial intestinal fluid. The samples were incubated statically at 37 °C. The bacterial solution (100 µL) was collected aseptically at 0 h (A_0_) and 3 h (A), spread onto MRS agar medium plates, and the colonies per plate counted. The experiment was performed with three replicates. The survival rate after simulated gastric or intestinal fluid treatment = (A/A_0_) × 100%, where A_0_ is the viable cell count (CFU/mL) at 0 h (before treatment) and A is the viable cell count (CFU/mL) after 3 h of treatment.

#### 2.4.5. Cell-Surface Hydrophobicity

The hydrophobicity of strain Y300 was determined with the method reported by Farid et al. [25]. The concentration of the bacterial solution was adjusted to 10^8^ CFU/mL (A_0_). The lactic acid bacteria were thoroughly mixed with xylene in a ratio of 1:1, shaken for 2 min (A), and then allowed to stand at room temperature for 2 h. Percentage (%) hydrophobicity = 1 − (A/A_0_) × 100%, where A_0_ is the initial optical density at 600 nm (OD_600_) before mixing with xylene, and A is the OD_600_ of the aqueous phase after phase separation.

#### 2.4.6. Determination of Auto-Aggregation and Co-Aggregation Abilities

Auto-aggregation and co-aggregation abilities were assessed according to [26,27]. Briefly, the concentration of the bacterial solution was adjusted to 10^8^ CFU/mL, and it was stored at 4 °C for later use. The initial OD600 (A_0_) of an aliquot (4 mL) of the bacterial suspension was measured. The suspension was then incubated at room temperature for 5 h, and the OD600 of a sample from the upper suspension was measured (A). Percentage (%) auto-aggregation = 1 − (A/A_0_) × 100%. In this formula, A_0_ represents the initial OD_600_ measurement, and A is the OD_600_ measured from the upper suspension after 5 h of incubation.

For the co-aggregation experiment, 2 mL of Y300 suspension (A_1_) was mixed with 2 mL of pathogenic bacterial suspension (*E. coli*, *Salmonella enterica Typhimurium*, *Salmonella enterica Pullorum*, *Salmonella enterica Enteritidis* or *S. aureus*) (A_2_), and incubated at 37 °C for 2 h. At the end of the incubation period, the OD600 was measured (A_3_). Percentage (%) co-aggregation = 1 − (A_3_/[A_1_+ A_2_]/2) × 100%,where A_1_ is the initial OD_600_ of the *Lpb. plantarum* Y300 suspension, A_2_ is the initial OD_600_ of the pathogenic bacterial suspension, and A_3_ is the OD_600_ of the mixed suspension after 2 h of co-incubation.

#### 2.4.7. Antioxidant Activity Analysis

The antioxidant activity of the strain Y300 was evaluated by determining its2,2-diphenyl-1-picrylhydrazyl (DPPH) radical scavenging ability as described by Zheng et al. [28] with slight modifications. The DPPH (free-radical)-scavenging ability of a sample of supernatant of the bacterial solution was measured with the DPPH Free Radical Scavenging Capacity Assay Kit (Solarbio, Beijing, China). The sample was incubated at 25 °C in the dark for 30 min, and the OD515 measured. Percentage (%) DPPH scavenging = 1 − (A − A_0_)/A_1_ × 100%, where A = experimental group (supernatant + DPPH); A_0_ = blank control (supernatant + water); and A_1_ = control group (culture medium + DPPH).

### 2.5. Analysis of the Complete Genomic Sequence of Y300

Genomic DNA was isolated with the GeneJET Genomic DNA Purification Kit (Thermo Fisher Scientific, Abingdon, UK), according to the manufacturer’s instructions. After DNA quality inspection and library construction, the sample library to be sequenced was constructed. After the filtering adapters, short fragments, and low-quality data were removed, data were acquired with a third-generation Nanopore sequencer and a second-generation Illumina sequencer. Unicycler (version 0.5.0) was used to assemble the second- and third-generation data. PlasFlow software (version 1.1.0–c85355d) was used to identify plasmids in the bacterial genome assembly results. BLAST software (version 2.11.0+) and the PLSDB database were used to annotate the plasmid sequences obtained. Prokka software (version 1.14.6) was used to predict the coding genes in the assembled genome. The antiSMASH program (version 6.0.0) was used to predict gene clusters encoding the components of secondary metabolite synthesis in the genome [29] and the bacteriocin production gene cluster was predicted with the Bagel4 database (http://bagel4.molgenrug.nl, accessed on 17 April 2025) [30]. Antibiotic resistance genes and genes encoding virulence factors were determined with the Comprehensive Antibiotic Resistance Database (CARD) [31] and the Virulence Factor Database (VFDB) [32], respectively. Functional annotation information on the protein sequences of the annotated coding genes was obtained with Gene Ontology (GO) [33] and the Kyoto Encyclopedia of Genes and Genomes (KEGG) [34]. Carbohydrate-active enzyme (CAZyme)-associated genes were identified in the Y300 genome with the dbCAN2 online server (https://bcb.unl.edu/dbCAN2/index.php, accessed on 17 April 2025) using a DIAMOND blast search in the CAZy database (http://www.cazy.org, accessed on 17 April 2025) [35].

### 2.6. Comparative Genomic 

Based on the whole-genome sequences of diverse LAB, the core gene *pheS* was selected, and a phylogenetic tree constructed with the maximum likelihood method (1000 bootstrap replicates; in MEGA 7) [36]. A multiple-sequence alignment was constructed from the protein sequences of the target strain and its close relatives. Subsequent analyses included gene family clustering, phylogenetic tree construction, and a collinearity analysis of the gene sequences from these species [37].

### 2.7. Genome Accession Number

The complete genomic sequence was submitted to the National Biotechnology Information Center (NCBI) GenBank database with the accession numbers PRJNA1321165.

### 2.8. Statistical Analysis

All the experiments were performed in triplicate and data are presented as means ± standard deviations. GraphPad Prism (version 10) was used to determine statistical significance with one-way ANOVA or a *t* test. *p* < 0.05 was considered to indicate statistical significance.

## 3. Results

### 3.1. Identification of Y300

**Figure 1:**
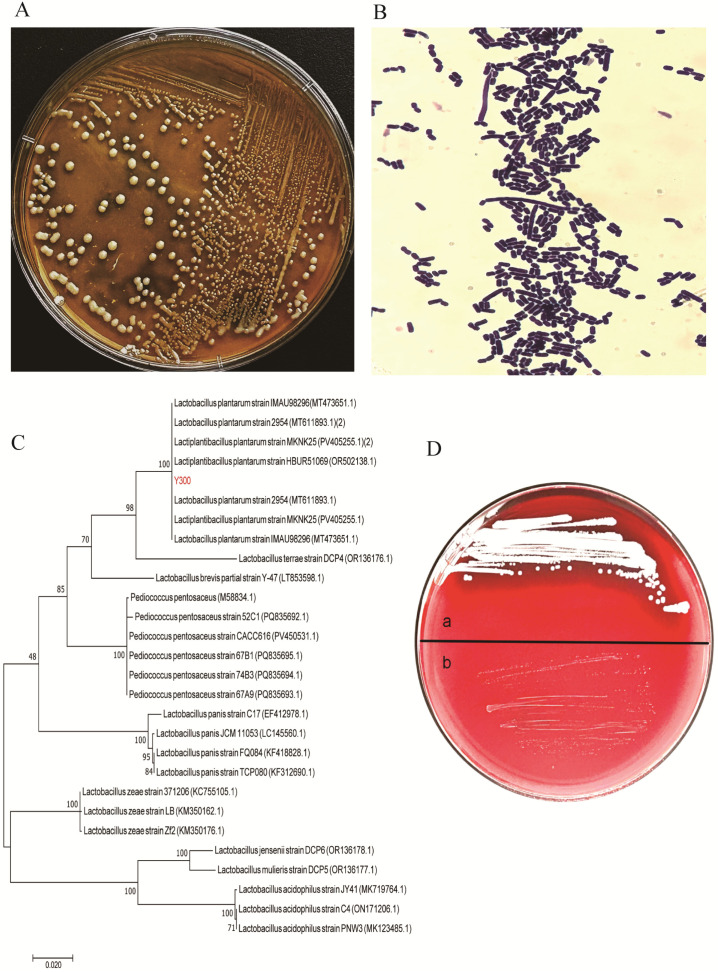
Isolation and preliminary characterization of *Lpb. plantarum* Y300. (**A**) Colony morphology of strain Y300 on MRS agar of incubation. (**B**) Gram staining results of strain Y300. (**C**) Phylogenetic tree based on 16S rRNA gene sequences, showing the evolutionary relationship between Y300 and related type strains. The tree was constructed using the neighbor-joining method, and bootstrap values (based on 1000 replicates) are shown at the nodes. Scale bar, 0.005 substitutions per nucleotide position. (**D**) Hemolytic activity analysis of *Lpb. plantarum* Y300 with blood agar plates. Y300 exhibited γ-hemolysis (no hemolysis), indicating a non-pathogenic trait. (a. *S. aureus* and b. Y300).

Strain Y300 formed convex, creamy-white colonies with a spherical form on MRS agar (Figure 1A). Microscopic examination following Gram’s staining revealed purple-colored, rod-shaped cells (coccobacilli), confirming the isolate as Gram-positive (Figure 1B). Physiological and biochemical experiments showed that strain Y300 was a lactic acid bacterium (Table S1). 16S rDNA sequencing and a DNA sequence alignment indicated that strain Y300 shared >99% identity with *Lactiplantibacillus*. A phylogenetic tree was constructed using MEGA 7 software, as shown in Figure 1C. Hemolytic activity was assessed on blood agar, where α-hemolysis is characterized by a greenish zone, β-hemolysis by a clear (translucent) zone, and γ-hemolysis by the absence of a zone. Strain Y300 was identified as non-hemolytic (γ-hemolytic), as no lysis zone was observed around its colonies (Figure 1D).

### 3.2. Antimicrobial Activity

**Figure 2:**
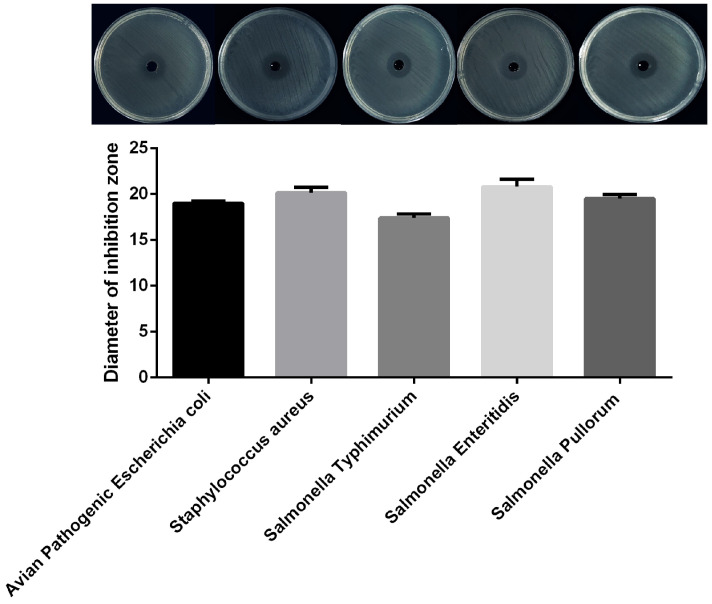
The inhibitory effects of *Lpb. plantarum* Y300 against pathogenic indicator bacteria.

The diameters of the zones of inhibition of the fermented supernatant of *Lpb. plantarum* Y300 against avian pathogenic *E. coli*, *S. aureus*, *Salmonella enteric Typhimurium*, *Salmonella enteric Enteritidis*, and *Salmonella enteric Pullorum* were 19 ± 0.25, 20.16 ± 0.6, 17.4 ± 0.45, 20.8 ± 0.81, and 19.5± 0.44 mm, respectively, indicating that *Lpb. plantarum* Y300 exerted significant inhibitory effects on all five pathogenic bacteria (Figure 2).

### 3.3. Antibiotic Susceptibility

Antibiotic susceptibility testing revealed that isolate Y300 was susceptible to the following antibiotics: erythromycin, penicillin, ceftriaxone, ampicillin, chloramphenicol, tetracyclines, and trimethoprim–sulfamethoxazole. The isolate was also resistant to ciprofloxacin and gentamicin (Table 1).

### 3.4. Growth and Acid Production Curves

**Figure 3:**
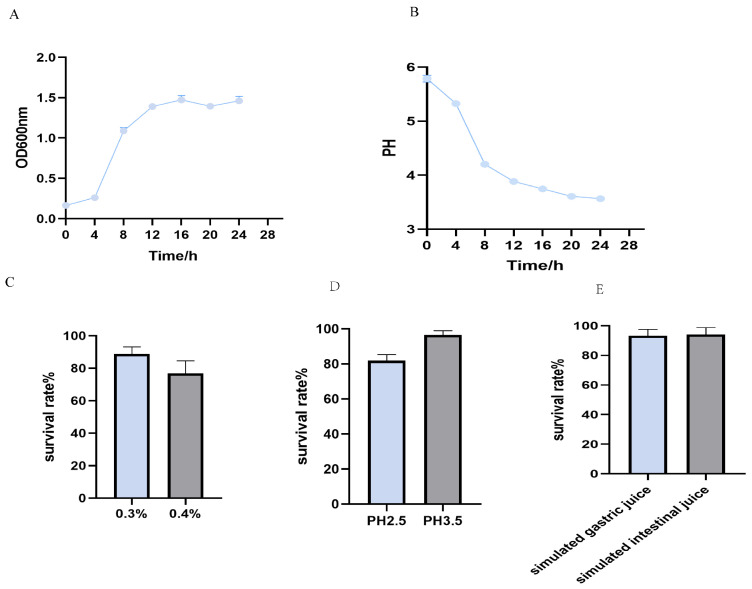
Growth, acid production, and stress tolerance of *Lpb. plantarum* Y300. (**A**) Growth curve of Y300 in MRS broth at 37 °C. Data points represent the mean ± SD of optical density at 600 nm (OD_600_) from three replicates. (**B**) Acid production curve, showing the corresponding decrease in pH of the culture medium over time. (**C**) Bile salt tolerance assessed by survival rate after exposure to 0.3–0.4% (*w*/*v*) porcine for 3 h. Y300 maintained a high survival rate. (**D**) Acid tolerance assessed by survival rate after exposure to pH 2.5–3.5 for 3 h. (**E**) Effect of simulated gastric and intestinal fluid on growth of *Lpb. plantarum* Y300.

*Lpb. plantarum* Y300 was in the lag period from 0 to 4 h, entered the exponential phase of growth after 4 h, and reached stationary phase at 16 h (Figure 3A). The strain also showed a strong capacity for acid production (Figure 3B).

### 3.5. Bile and Acid Tolerance

Strain Y300 was assessed for bile and acid tolerance. The results showed a survival rate of 92% in 0.3% bile salt and 80% in 0.4% bile salt (Figure 3C). The effect of acidity on the viability of Y300 strain was also investigated. The isolate showed tolerance of low pH values (Figure 3D).

### 3.6. Tolerance of Simulated Gastric and Intestinal Fluids

Strain Y300 showed excellent gastrointestinal tolerance, with a survival rate of 93.1% after treatment with simulated gastric fluid and 94% in simulated intestinal fluid (Figure 3E).

### 3.7. Probiotic Surface Properties

**Figure 4:**
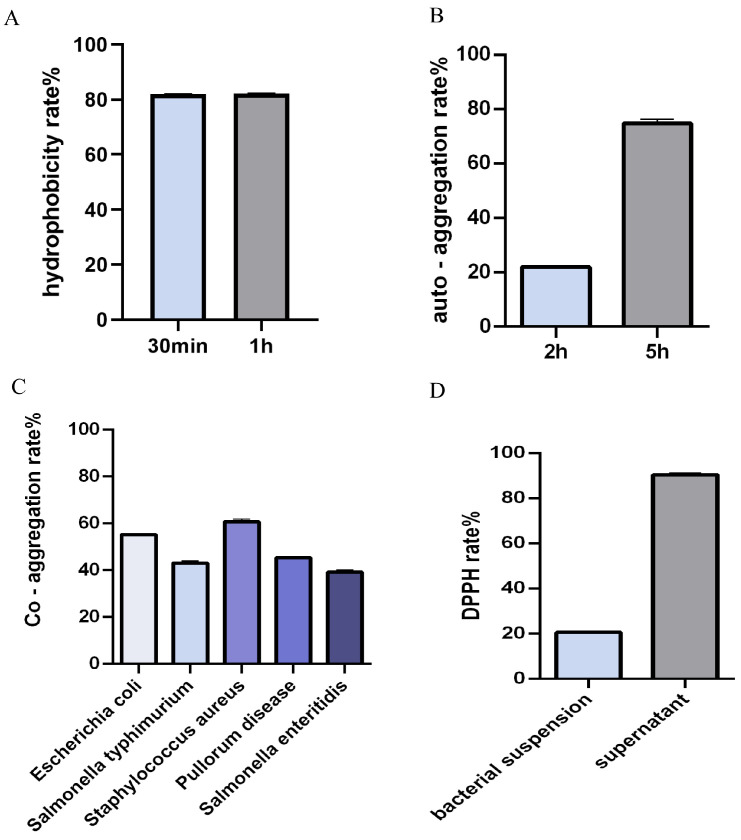
Cell surface properties and antioxidant activity of *Lpb. plantarum* Y300. (**A**) Cell surface hydrophobicity assessed by microbial adhesion to hydrocarbons (xylene). (**B**) Auto-aggregation ability measured. Y300 showed a high auto-aggregation percentage of 74.72% at 5 h. (**C**) Co-aggregation ability of Y300 with pathogenic bacteria after 2 h of co-incubation. (**D**) Antioxidant activities of *Lpb. plantarum* Y300 analyzed by radical scavenging activities tests.

The hydrophobicity of strain Y300 was examined with xylene. Strain Y300 displayed relatively high surface hydrophobicity, with a hydrophobicity rate of 67.7% (Figure 4A). A self-aggregation assay indicated that the auto-aggregation rates of strain Y300 in phosphate-buffered saline (PBS) at 2 h and 5 h were 21.95% and 74.72%, respectively (Figure 4B). Generally, an auto-aggregation rate exceeding 50% is considered to meet the requirement for a probiotic. In a co-aggregation assay, strain Y300 showed strong co-aggregation ability with five pathogens (Figure 4C). The antioxidant performance of strain Y300 was investigated with the free radical DPPH. A Y300 bacterial suspension had a DPPH-scavenging rate of only about 20%, whereas the scavenging rate of the fermented supernatant was 90.45%, indicating that the fermented supernatant of Y300 had good free-radical-scavenging ability (Figure 4D).

### 3.8. Complete Genomic Sequencing and Bioinformatic Analysis

**Figure 5:**
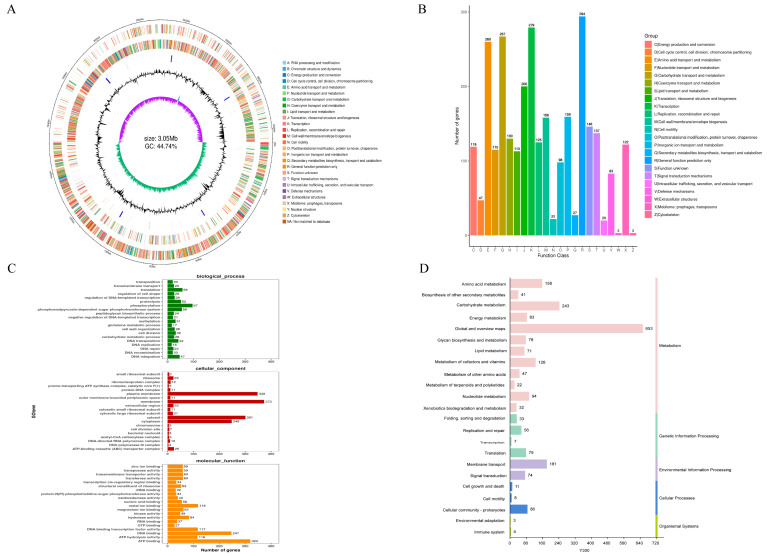
Genomic features and functional annotation of *Lpb. plantarum* Y300. (**A**) Circular draft genome map of *Lpb. plantarum* Y300. (**B**) COG database classification statistics. (**C**) GO term distribution across biological process, cellular component, and molecular function. (**D**) KEGG pathways of proteins functional.

Whole-genome sequencing showed that the Y300 genome is circular (3,054,295 bp, GC content 44.74%); contains 3158 genes; and encodes 63 tRNAs, five 23S rRNAs, five 16S rRNAs, six 5S rRNAs, one tmRNA, 47 misc_RNAs, and eight CRISPR sequences (Figure 5A). Y300 also had 2513 Clusters of Orthologous Groups (COG) functional annotations (Figure 5B), primarily enriched in function prediction (Class R), translation and ribosome biogenesis (Class J), transcription (Class K), amino acid transport and metabolism (Class E), and carbohydrate transport and metabolism (Class G). GO annotations showed that most genes were enriched in cellular components and molecular functions, with genes mainly expressed in cell membranes, the cytoplasm, and the periplasmic space. The genes enriched in molecular functions were mainly involved in ATP binding/hydrolysis, DNA binding, transcription factor activity, and metal ion binding (Figure 5C), indicating their involvement in the complex cellular metabolism and diverse molecular functions. The KEGG pathways were mainly enriched in carbohydrate metabolism, amino acid metabolism, cofactor/vitamin metabolism, phosphotransferase system, ABC transporters, and bacterial secretion systems (Figure 5D).

### 3.9. Identification of CAZymes

**Figure 6:**
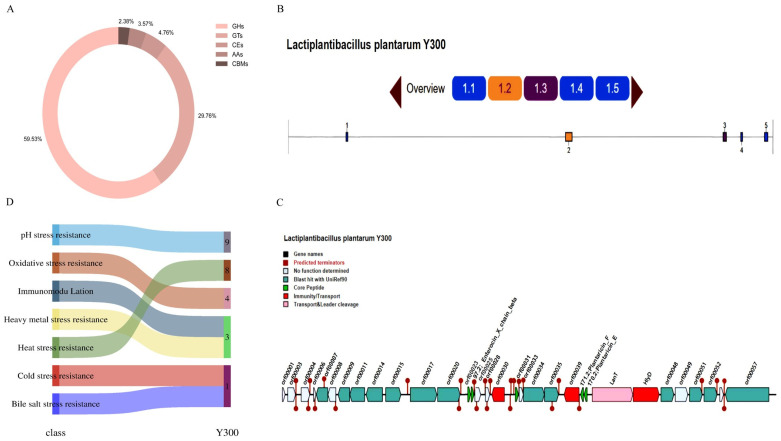
In silico analysis of probiotic potential genes in Y300 genome. (**A**) CAZyme family distribution. (**B**) Secondary metabolite cluster predicted as Class II bacteriocin by antiSMASH. (**C**) BAGEL4 prediction of bacteriocin gene cluster containing lagD. (**D**) Statistical annotation of databases encoding potential probiotic genes.

The functional annotation of the Y300 genome revealed genes involved in carbohydrate metabolism and biosynthesis, including glycoside hydrolases (GHs), glycosyl transferases (GTs), carbohydrate esterases (CEs), auxiliary activities (AAs), and carbohydrate-binding modules (CBMs). Specifically, the genome encoded 50 GHs, 25 GTs, 4 CEs, 3 AAs, and 2 CBMs (Figure 6A).

### 3.10. Identifications of Gene Clusters Encoding Secondary Metabolites and Bacteriocin

The analysis of the whole Y300 genome sequence with antiSMASH 7.1.0 identified four gene clusters involved in the biosynthesis of secondary metabolites on its chromosome: RiPP-like, T3PKS, terpene, and cyclic-lactone-autoinducer (Figure 6B). Among these, the RiPP-like gene cluster included the core biosynthetic gene *lagD*, which encodes lactococcin G. Further validation with the BAGEL4 database successfully located the bacteriocin synthesis gene cluster related to lactococcin G in the Y300 genome (Figure 6C).

### 3.11. Identification of Antibiotic Resistance Genes and Virulence Genes

The coding genes of Y300 were compared with CARD using a screening criterion of >99% identity, and no resistance genes were found. The coding genes of Y300 were compared with the VFDB database using a percentage of identical matches (pident) >90% as the screening criterion, and no virulence genes were detected.

### 3.12. Stress-Resistance Genotype Analysis

Analysis of the genomic sequence of Y300 identified several genes involved in resistance to adverse environmental stresses (Figure 6D), including pH stress resistance genes (*atpF*, *atpB*, *atpE*, *atpD*, *atpA*, *atpG*, *atpH*, *atpC*, and *cadA*), bile salt stress resistance genes (*ppaC*), antioxidant genes (*trxA-2*, *msrA-1*, *msrB*, and *mntH-3*), heavy metal stress resistance genes (*copA*, *znuB*, and *znuC*), heat resistance genes (*clpB*, *clpC-2*, *grpE*, *hrcA*, *hslO*, *hslV*, *dnaK*, and *dnaJ*), cold resistance genes (*cshB*), and immune regulation genes (*dltA*, *dltC-1*, and *dltD*).

### 3.13. Comparative Genomic Analysis

**Figure 7:**
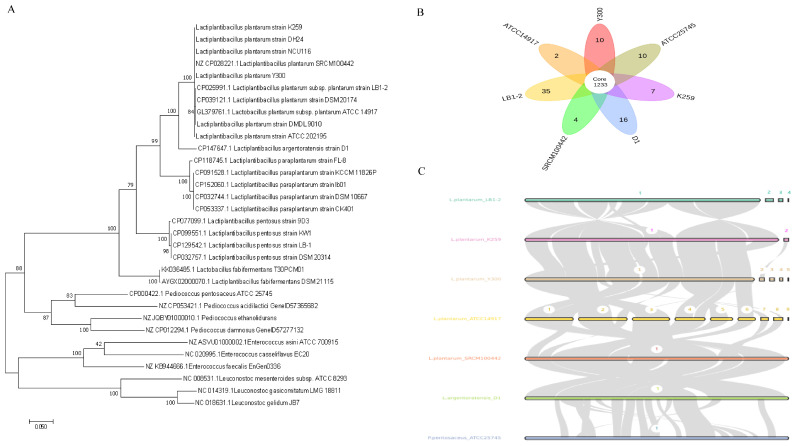
Comparative genomics of *Lpb. plantarum* Y300. (**A**) Phylogenetic tree of the strain Y300 based on the *Phes* gene. (**B**) Venn diagram showing the common genes and unique genes in Y300 and 6 other *L. plantarum*. (**C**) Collinearity block global sequence alignment between *Lpb. plantarum* Y300 and another 6 strains of *Lpb. plantarum*. The numbers in the figure represent the proportion of syntenic blocks, which was derived from a whole-genome collinearity analysis.

A phylogenetic tree was constructed using the core gene *pheS*, based on the whole-genome sequencing results. *Lactiplantibacillus plantarum* Y300 clustered with *Lpb. plantarum* strains NCU116, K259, and DH24 on the same evolutionary branch (Figure 7A). A clustering analysis showed that seven LAB from different sources, including Y300, shared 1233 core gene families. Y300 had 10 unique gene families (containing 30 genes), including a functional gene encoding the acidic shock protein Hsp20 (ctg_03094, ctg_03095) (Figure 7B). A collinearity analysis was used to examine the consistency of homologous sequences and their order among different species and strains to evaluate their genomic relatedness. The Y300 genome showed high conservation of its homologous gene arrangement with the closely related *Lpb. plantarum* strain K259, but also gene rearrangements (such as inversions and translocations). In contrast, the genomic structure of Y300 differed significantly from that of ATCC14917 (Figure 7C).

## 4. Discussion

The search for sustainable alternatives to antibiotics in poultry farming has increasingly focused on probiotics as a viable strategy [38]. A prevailing rationale favors probiotics isolated from the host species, based on the premise that such strains possess superior ecological fitness, survival rates, and functional efficacy within the native gut environment [39]. These indigenous microorganisms are integral to sustaining a balanced gut microbiome, which is fundamental to overall animal health [40].Because the intestinal contents and feces are the primary sources of probiotics [41], in this study, we screened cecal contents for probiotics to identify novel probiotic strains.

Antimicrobial activity is a key characteristic in probiotic screening. Metabolites produced by lactic acid bacteria, such as bacteriocins, organic acids, and exopolysaccharides, can inhibit pathogens in the gut, prevent pathogen colonization, maintain the stability of the gut microbiota, and prevent or alleviate diseases caused by pathogens. They also contribute to the maintenance of the intestinal epithelial barrier and modulate the host immune responses [42,43]. In our screening, the chicken intestinal isolate *Lpb. plantarum* Y300 exhibited potent antagonistic activity against key pathogens, including *E. coli*, *S. enterica*, and *S. aureus*. This broad-spectrum inhibition is likely mediated by bacteriocins, which are ribosomally synthesized antimicrobial peptides known for their stability. The putative bacteriocins from Y300 may target fundamental cellular processes in pathogens, such as compromising membrane integrity, impeding cell wall assembly, and disrupting essential biosynthetic pathways [44].

After entering the host intestine, probiotics must typically colonize the intestine and proliferate rapidly to gain a competitive advantage in the microbial community and become the dominant microorganisms [45]. In this study, *Lpb. plantarum* Y300 entered the logarithmic growth phase after 4 h and reached the stationary phase within 16 h. In contrast, the chicken-derived LAB R26 reported by Tian et al. [16] entered the logarithmic phase after 10 h and reached the plateau phase at 18 h, indicating that Y300 exhibits a superior growth rate compared to R26. Y300 also showed a strong acid-producing capability, which would maintain a suitable environment and inhibit the growth of harmful bacteria.

The ability of probiotics to withstand various adverse factors in the gastrointestinal tract (digestive enzymes, bile salts, low pH, gastric acid, etc.) is crucial for their colonization of and proliferation within the host intestine. Therefore, such tolerance is a primary requirement when screening and evaluating probiotics [46]. Based on previous studies, pH 2.0–3.0 [47] and 0.3% bile salts [27] are commonly used as the thresholds for testing the acid tolerance and bile salt tolerance of potential probiotics, respectively. Tang et al. [48] reported that the MA2 showed a survival of 70% at pH 2.5 and 0.3% bile salt for 3 h. In another study, six LAB strains isolated from chickens showed good tolerance to 0.3% bile salt after 6 h of exposure and moderate-to-good survival in simulated gastric juice with a pH of 2.0 [27]. In this study, isolated strain Y300 showed strong tolerance of high bile salt concentrations, low pHs, and simulated gastrointestinal fluids for 3 h. Specifically, Y300 showed survival rates of 88.86–93% at a bile salt concentration of 0.3% and 71–80% at a concentration of 0.4%. Its survival rates in simulated gastric fluid and simulated intestinal fluid were 89.33–96.81% and 84.94–96.39%, respectively. These survival rates were significantly higher than those recently reported for other poultry-derived LAB, indicating that Y300 is a promising candidate probiotic strain.

Probiotics exert their beneficial effects by inhibiting pathogen invasion, improving the intestinal epithelial barrier function, and modulating the host’s immune system [49]. Therefore, the ability of probiotics to adhere to and colonize the gastrointestinal tract is a prerequisite for their efficacy. Surface property tests (auto-aggregation, hydrophobicity) are commonly used to assess probiotic adhesion [50]. Generally, probiotics with high hydrophobicity values show stronger adhesion to epithelial cells [51]. Previous studies have reported hydrophobicity values ranging from 40.5% to 71.0% for lactic acid bacterial strains isolated from poultry [27]. According to studies by Roggeman and McGreal, a strain must have an auto-aggregation rate exceeding 40% to be considered a potential probiotic [52]. In the present study, strain Y300 showed 55.37–71.06% hydrophobicity and 40–54.66% auto-aggregation, demonstrating good potential adhesion. Probiotics are natural antioxidants, and their antioxidant activity alleviates the oxidative stress caused by an imbalance in the reactive oxygen species or free radicals in the body, thus slowing the pathological changes induced by oxidative damage [53]. In this study, we used DPPH to evaluate the antioxidant performance of Y300, which showed a good DPPH-scavenging rate.

Combining multiple evaluation methods allows a more comprehensive assessment of probiotic properties than the use of a single method. Beyond phenotypic characterization, whole-genome sequencing permits the analysis of a strain’s complete genetic information at the gene level, offering a rapid and accurate method of evaluating a probiotic’s function and safety [54]. In this study, sequencing the whole genome of strain Y300 revealed a genome size of 3.05 Mb, consisting of a single chromosome with a G + C content of 44.74%. These parameters differ from those of *Lpb. plantarum* strains from other sources, indicating high variability among different lactobacilli, which allows them to adapt well to their specific environments [48].

CAZymes play a key role in the host’s nutrient metabolism. The Y300 genome contains 84 CAZyme genes, encoding 50 GHs, 25 GTs, four CEs, three AAs, and two CBMs. Notably, numerous genes of Y300 encode GHs and GTs. These enzymes catalyze the transfer of sugars to specific receptors and play critical roles in forming the surface structures recognized by the host immune system. GHs catalyze the cleavage of glycosidic bonds, releasing abundant energy and helping the host resist the invasion of and adhesion to the intestinal epithelium by potential pathogens and their toxins [55,56]. While a high CAZyme count is a common feature in *Lpb. plantarum*, the specific profile of Y300 may reflect an adaptation to its unique niche in the free-range chicken cecum. Future studies should focus on correlating this genetic potential with the actual utilization of specific carbohydrate sources.

From a safety and application perspective, the genome of Y300 harbors desirable traits. The presence of a CRISPR-Cas system is of particular interest, as it not only provides a defense mechanism against phage predation, but also inherently limits the horizontal gene transfer (HGT) of antibiotic resistance genes [57]. This finding significantly bolsters the safety profile of Y300, as it reduces the risk of disseminating resistance determinants in the gut microbiome. Complementing this, the identification of a bacteriocin gene cluster, specifically the *lagD* gene involved in lactococcin G production, provides a direct molecular explanation for the observed antimicrobial activity against pathogens like *Salmonella* and *E. coli* [44]. The synergistic action of inhibiting pathogen growth (via bacteriocins) [58] and limiting HGT (via CRISPR-Cas) positions Y300 as a highly competitive and safe probiotic candidate, a combination that is increasingly sought after but not always found in potential probiotic strains.

16S rRNA gene sequences cannot reliably distinguish *Lpb. plantarum* from closely related species, whereas the sequence of housekeeping gene *pheS* effectively differentiates *Lpb. plantarum* from its close relatives [59]. Therefore, we used the *pheS* sequence from the Y300 genome for the intraspecific typing of *Lpb. plantarum*. A comparative genomic analysis with six *Lpb. plantarum* strains from different sources revealed that the Y300 genome contains unique gene families, including the *Hsp20* gene (ctg_03094, ctg_03095), which encodes an acidic shock protein function. This gene allows bacteria to survive and maintain normal functions under acid stress and reduces bacterial oxidative damage [60], indicating that strain Y300 has a strong capacity to adapt to the complex gastrointestinal environment. A collinearity analysis, which detects the consistency of homologous sequences and their order across the genomes of different species or strains [61], was used to study the genomic relatedness of Y300, and showed significant collinearity between the Y300 strain and the kimchi-derived *Lpb. plantarum* K259 genome. This analysis revealed a highly conserved core gene arrangement, suggesting that *Lpb. plantarum* has maintained the genetic basis for its key life activities during its differentiation, with no large-scale chromosomal rearrangements or horizontal gene transfer. This implies strong consistency in the core probiotic functions (acid and bile salt tolerance, adhesion) of these two strains, supporting their interchangeable application across sources.

Given the widespread use of probiotics in the poultry industry, the safety of probiotic strains is a crucial screening criterion [62]. Because hemolytic strains can cause sepsis in the host, nonhemolytic bacteria are considered essential as quality probiotics [46,63]. Y300 showed no hemolytic properties on blood agar plates. To mitigate clinical risks, it is essential to systematically evaluate the antimicrobial susceptibility of probiotics, as they may harbor intrinsic or acquired antibiotic resistance genes. The intrinsic resistance to ciprofloxacin and gentamicin, common in lactobacilli due to the impermeability of their cell membranes, is generally not considered transmissible. Our genomic analysis confirmed the lack of known acquired antibiotic resistance genes, aligning with the guidelines set by EFSA and WHO for the safety evaluation of probiotics [64]. Furthermore, no virulence genes were detected in the Y300 genome. These findings collectively demonstrate the safety of strain Y300 and support its potential use as a probiotic.

## 5. Conclusions

We isolated *Lpb. plantarum* Y300 from locally raised, free-range native chickens. It displayed outstanding comprehensive features: potent broad-spectrum antimicrobial activity, exceptional gastrointestinal tolerance (acid and bile salt resistance), excellent adhesion and antioxidant capabilities, and rapid growth with strong acid production. The genomic insights were pivotal in elucidating the mechanistic basis of its probiotic traits: the CAZyme repertoire predicts nutrient metabolism capabilities, the bacteriocin genes explain its antimicrobial activity, and stress-response genes underpin its gastrointestinal tolerance. Most importantly, the absence of detected virulence or transferable resistance genes in the genome solidifies its safety case beyond phenotypic assays. These findings not only recommend Y300 for use as a feed additive to enhance poultry health and production but also, given its genetic makeup, position it as an ideal candidate for developing targeted synbiotic products and exploring its efficacy as a biotherapeutic agent in livestock management.

## Figures and Tables

**Table 1:** Antibiotic susceptibility of *Lpb. plantarum* strain Y300.

Class of Antibiotic	Antibiotic		R	I	S	Zone of Inhibition in Millimeters (mm)
Macrolide antibiotics	Erythromycin	8	≤13	14–22	≥23	32.8 ± 0.5 (S)
beta-lactam antibiotics	Penicillin	8	≤28	-	≥29	28.9 ± 0.7 (S)
	Ceftriaxone		≤22	23–25	≥26	34.6 ± 0.8 (S)
	Ampicillin	8	≤13	14–16	≥17	30.85 ± 0.75 (S)
Fluoroquinolones	Ciprofloxacin		≤15	16–20	≥21	7.5 ± 0.5 (R)
Aminoglycosides	Gentamicin		≤12	13–14	≥15	10.3 ± 2.3 (R)
Chloramphenicol	Chloramphenicol		≤12	13–17	≥18	26.8 ± 0.7 (S)
Tetracyclines	Tetracycline		≤11	12–14	≥15	21.05 ± 0.15 (S)
Sulfonamides	Trimethoprim- Sulfamethoxazole		≤10	11–15	≥16	20.85 ± 0.65 (S)

## Data Availability

The original contributions presented in this study are included in the article/Supplementary Material. Further inquiries can be directed to the corresponding authors.

## Supplementary Materials

The following supporting information can be downloaded at: https://www.mdpi.com/article/10.3390/microorganisms13122738/s1, Table S1: Biochemical characterization of *Lpb. plantarum* Y300.

## Abbreviations

The following abbreviations are used in this manuscript:

S	Susceptible
I	Intermediate
R	Resistant
