# Supplementary material for: Complete Genome Sequence Analysis and Probiotic Characterisation of Lactiplantibacillus plantarum Y300 Isolated from Traditional Free-Range Chickens
Source: Microorganisms. 2025 Nov 30;13(12):2738. doi: 10.3390/microorganisms13122738 (PMC12735756; doi:10.3390/microorganisms13122738)
Supplement: Supplementary file 1 [file microorganisms-13-02738-s001.zip › microorganisms-3982802-supplementary.pdf]

Table S1. Biochemical characterization of *Lpb. plantarum* Y300.

**Test**	**Result**
ESCULIN HYDRATE	+
CELLOBIOSE	+
D-MALTOSE	+
D-MANNITOL	+
SALICIN	+
D-SORBITOL	+
SACCHAROSE/SUCROSE	+
D-RAFFINOSE	-
INULIN	+
LACTOSE	+
FRUCTOSE	+
D-RHAMNOSE	+
L-ARABINOSE	+
D-XYLOSE	+
D-GLUCOSE	+

Note: "+" indicates positive; "-" indicates negative.
